# Passive Shimming of MRI Static Magnetic Field Using Regularization of Truncated Singular Value Decomposition

**DOI:** 10.2463/mrms.mp.2016-0046

**Published:** 2017-03-02

**Authors:** Mitsushi Abe

**Affiliations:** Hitachi, Ltd., Research & Development Group, Center of Technology Innovation–Energy, 7-2-1 Omika-cho, Hitachi, Ibaraki 319-1221, Japan

**Keywords:** magnetic resonance imaging, magnetic field shimming, homogeneity, singular value decomposition, homogeneous magnetic field

## Abstract

**Purpose:**

To develop a new shimming calculation method, which can calculate iron piece placements rapidly to make the magnetic field homogeneous at intended homogeneity and then to make the shimming working time short.

**Materials and Methods:**

The shimming calculation yields magnetic moment (MM) distribution, which is calculated by the truncated singular value decomposition (SVD) from the measured magnetic field. The MM distribution is described by a superposition of eigenmodes obtained by SVD of a response matrix from the moment distributions to magnetic fields at the field of view (FOV). The homogeneity is regulated by a truncation number of the superposed eigenmodes. The magnetic moments are converted into iron volumes with the assumption of saturated magnetization and the iron pieces are placed according to the calculation results. Since the SVD calculation can be done in advance, the computational time at the shimming site is short.

**Results:**

Trial applications on a 0.5T magnetic resonance imaging (MRI) magnet were done using the new shimming calculation method, which was proved to work well. However, since the iron piece volumes had tolerances, the work was repeated until enough homogeneity was obtained. As a result, an intended homogeneity of 8.9 ppm (peak-to-peak) on 40 cm diameter spherical surface was successfully obtained from measured homogeneity of 543 ppm with short computational and working time.

**Conclusion:**

The test shimming work showed that the developed shimming calculation method with truncated SVD regularization is applicable to the shimming work on the MRI magnets.

## Introduction

Magnetic resonance imaging (MRI) magnets have strong and homogeneous static magnetic fields at their field of view (FOV) to get clear imaging. The static magnetic field homogeneity is defined by the amplitude of the magnetic field strength distribution divided by a mean magnetic field on the surface of the volume of interest (VOI), which is roughly equal to FOV usually and a 40 cm diameter spherical volume (40 cm-DSV) in this paper. The homogeneity in FOV should be on the order of 10 ppm peak-to-peak (PP),^1,2^ where ppm means parts per million (1 × 10^−6^). This study treats static magnetic field shimming. After this, the magnetic field is static one, the shimming means static magnetic field shimming and all homogeneities are described by ppm simply, meaning they are peak-to-peak values in 40 cm-DSV.

MRI Magnets are designed with adequate coil block (CB) placements to have the capabilities to produce sufficient homogeneities.^3–5^ However, the magnets have some errors in their static magnetic fields due to manufacturing tolerances on CB placements and magnetic site environment. Each just manufactured magnet is subjected to a magnetic field correction procedure called shimming work to ensure they have a designed homogeneous magnetic field at the installation site.^1,2^ The shimming work makes homogeneity from several hundred ppm to the designed value which is on the order of 10 ppm over FOV.^2^

There are two kinds of shimming techniques. One is passive one and the other is active one.^1,2^ The passive shimming commonly uses iron pieces as shims. The iron pieces are magnetized passively due to the strong magnetic field and the magnetized iron pieces have magnetic moments (MMs) which generate a magnetic field to correct error fields.^6–13^ The active shimming uses small coils (shim-coils) and many shim-coils are necessary for accurate shimming with an increased magnet cost. However, the shim-coils for the active shimming can also be considered to have MMs by applied currents, and the MMs generate correcting magnetic fields. This situation is same as that of the passive shimming.

If MM placements are known, the magnetic field can be well calculated. However, for the shimming, the placements have to be calculated to compensate the error magnetic field, which is a distribution of the deviation between the homogeneous target magnetic field and the measured magnetic field. This is a shimming calculation, which is an inverse problem and is the subject of this study. This study treats the passive shimming and the calculated MM distribution is converted into iron piece placements.

Some shimming calculation methods have been proposed. One uses linear optimization (LO) to search iron piece placements which minimize error magnetic field,^6^ and others use constraints such as iron volumes.^7,8^ Finally, some methods shim the magnetic fields through eliminating the coefficients of spherical harmonic functions (SHFs).^9–13^

These methods have yielded successful shimming works for cylindrical MRI magnets. However there are two concerns. One is a computational time. The shimming works on the cylindrical MRI magnets, are done with ramp-down and ramp-up of the magnetic fields in a few hours, meaning that one hour computational time does not disturb the shimming works. However, the shimming calculation in this paper is to be applied to the open MRI magnets, which shimming works can be done without the ramp-down and ramp-up, meaning that the computational time should be less than the magnetic field measurement time and it should a few minutes or less. Once we tried the LO and found that it took a few tens of minutes. Then, the shimming calculation using LO is considered to be not suitable for the shimming work of the open MRI. Another concern is about the uses of SHFs for which the shimming is done by placing MMs (iron pieces) on planar or cylindrical shim-trays. The SHFs form bases of the VOI surface magnetic fields. However, there are no corresponding bases on the shim-trays. Then, the functions for MM placements become complicated. This fact means that eliminating low ordered SHFs may cause some error fields at high ordered SHFs, which disturb the shimming and deteriorate the homogeneity.

Usually, the shimming calculation has been considered to be a complicated problem. However, since the iron pieces are at near saturated magnetization, the shimming calculation can be solved as a system of linear equations, which describe relations between MMs and magnetic fields. An inverse calculation determines the MM placements from the error magnetic fields and the calculation needs a regularization.^14^

The same kind of the problem is found in nuclear fusion device designs, and DUCAS^15^ was developed to handle it. DUCAS calculates current potential^16^ (CP) or MM density distributions on arbitrary surfaces using a regularization of the truncated singular value decomposition^17^ (TSVD). The CP is identical to the flow function which is used for gradient field coil (GC) designs.^18,19^ DUCAS has also been applied to GC designs.^20,21^

In this study, DUCAS treats the MM placements as shims with continuous distributions like the magnetization mapping approach for shim designs.^7^ However, This novel method calculates CP distributions by DUCAS and CP values are converted into MM distributions and iron piece placements. This is possible because a CP value means that the current is flowing around the node^19^ and can be recognized as an MM density. For a short computation time in the shimming site, our method calculates singular value decomposition (SVD) in advance.

SVD has been applied to CB placements in MRI magnet design,^3,4^ which are done by tuning the large singular value (low ordered) eigenmodes. Such eigenmodes have significant magnetic field strengths. The low ordered eigenmodes are easy to have error magnetic fields^4^ and this novel shimming method tunes the low ordered eigenmodes, compatibly. The method worked well at test shimming works. It has been proved that our novel shimming calculation provides adequate iron piece placements for the shimming and good predictions of the shimmed magnetic fields from the measured magnetic fields.

This paper describes formulations of the shimming calculation method using TSVD regularization and results of shimming tests on a real MRI magnet.

## Materials and Methods

### Shimming geometry and procedure

**Fig 1.**
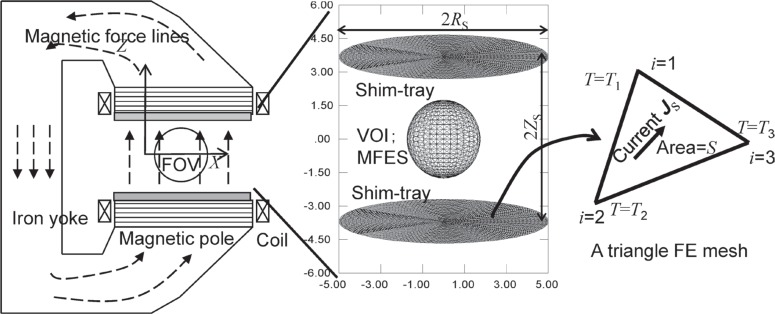
Schematic drawing of a magnet for an open type magnetic resonance imaging (MRI) and a shimming computational model. Left frame shows an open MRI magnet. Center frame shows a computational model with top and bottom shim-trays, and with magnetic field evaluation surface (MFES) at middle. In the shimming calculation, the shim-trays are formed by finite elements (FEs), one of which is shown in right with node current potentials. FOV, field of view; VOI, volume of interest.

Figure 1 is a schematic drawing of a geometry for a magnetic field shimming with a magnet, which is for open type MRI and has poles (top and bottom), between which is a homogeneous magnetic field with mainly vertical (*Z*) component. The poles have CBs and iron yokes. MRI magnets are used with radio frequency antennas and GCs, but they are not shown in the figure. The FOV is at the center of the magnet as shown by the circle and the VOI, in which magnetic field is shimmed, is roughly same as FOV. Passive shimming is applied with iron pieces.

**Fig 2.**
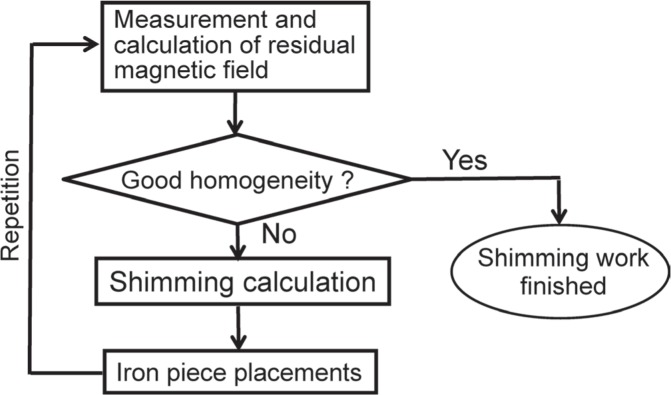
Procedure of the repetitive shimming work.

The shimming work is done repetitively with a procedure shown in Fig. 2. The work starts with magnetic field measurement. If the measured magnetic field meets the homogeneity specification, no further work is necessary. If the homogeneity is not sufficient, the shimming work continues until the homogeneity becomes good (meets the specification). The procedure after measurement is that the shimming calculation and the iron piece placements.

This procedure shims the magnetic field at magnetic field evaluation points (MFEPs) which are on the magnetic field evaluation surface (MFES). In this study, the MFES is the surface of the VOI. Magnetic fields on the MFES are measured and shimmed by the shims (iron pieces) on the shim-trays which are placed surrounding the VOI. Since the magnetic field is strong (a few 1/10 to several T), the iron pieces are at saturated magnetization in the magnetic field direction and, similarly, the MMs of the iron pieces are in the vertical (+*Z*) direction. The magnetic field in the FOV is almost completely aligned in the *Z* direction.

### Magnetic field

The shimming calculation is formulated with only the *Z* component *B*_Z_ and the target magnetic field is a uniform one at strength *B*^0^ in the FOV and we define the error field distribution ***B***^ER^ as,

(1) $$B^{\text{ER}}=B^{0}-B^{\text{MS}},$$

where ***B***^MS^ is a distribution vector of present measured magnetic field, and the components are *B*_Z_ strengths at MFEPs. There are several hundred magnetic field measurement points (MFMPs) and MFEPs. Generally MFMPs and MFEPs can be different each other. However, we define the MFEPs at the same positions as the MFMPs. They are on the MFES (VOI surface) of Fig. 1. The magnetic fields (component of the magnetic field vectors) in Eq. (1) are at MFEPs. They form vectors of, ***B***^ER^ = (-----, $B_{\text{i}}^{\text{ER}}$, ------), ***B***^0^ = (-----, $B_{\text{i}}^{0}$, -----) and ***B***^MS^ = (------, $B_{\text{i}}^{\text{MS}}$, ------) and each point *i* is at MFEP.

The shimming work is to produce a magnetic field ***B***^MM^ = (-------, $B_{\text{i}}^{\text{MM}}$, -----) at MFEPs by MMs of the iron pieces and ***B***^MM^ compensates ***B***^ER^. We assume here that MMs are distributed on the shim-trays as ***m***(***x***) [A], which is the magnetic moment [Am^2^] per an area [m^2^] and components are for *X*, *Y* and *Z* directions. The magnetic field at the *i*-th MFEP due to the MM at the *j-*th position ***M***_j_ = ***m***(***x***_j_)Δ *S*_j_ is,

(2) $$B_{\text{ij}}^{\text{MM}}=\left\{\mu_{0}/\left(4\pi r_{\text{ij}}^{3}\right)\right\}{\left[3\left(M_{\text{j}}\cdot r_{\text{ij}}\right)r_{\text{ij}}/r_{\text{ij}}^{2}-M_{\text{j}}\right]}_{\text{Z}},$$

where ***r***_ij_ is a vector from the *j*-th position to the *i-*th MFEP, *r*_ij_ is an absolute length of ***r***_ij_, μ_0_ is permeability in a vacuum and the last subscript *Z* means the *Z* component of the magnetic field.

**Fig 3.**
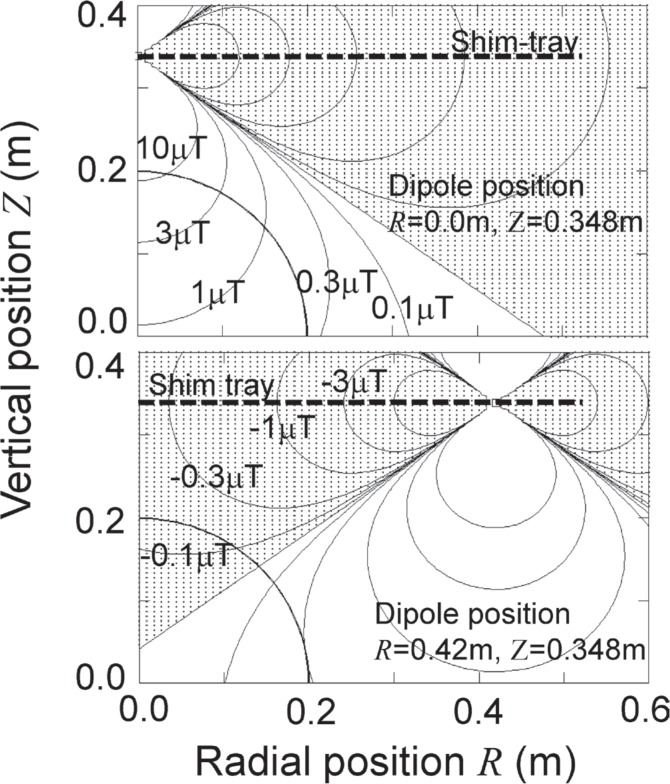
Magnetic field *B*_Z_ distribution by a magnetic dipole moment *M*_Z_ = 0.1711 Am^2^ (referenced to a 0.1 cc iron piece) placed. 1/4 circles at left bottom in each frame represent 40 cm-DSV surfaces.

Figure 3 plots the magnetic field distribution of Eq. (2). An iron piece of 0.1 cm^3^ with magnetization in *Z* direction (*M*_Z_ = 0.1711 Am^2^) is placed on the top shim-tray in each frame, changing the radial position at the center and edge. The iron piece strengthens the magnetic field in the VOI. However, when the piece is placed at the edge, it has a negative magnetic field (dotted area). On the top frame, the piece generates a magnetic field of more than 10 μT. which corresponds to 6.7 ppm and 20 ppm for *B*^0^ of 1.5T and 0.5T, respectively. This means that smaller pieces than 0.05 cm^3^ are necessary for accurate shimming to make the homogeneity down to 10 ppm in 40 cm-DSV on a 0.5T magnet. The shimming calculation in this study calculates the ***m***(***x***_j_) distribution which generates ***B***^MM^ roughly equal to ***B***^ER^.

### Formulation of shimming calculation

#### Formulation

**Fig 4.**
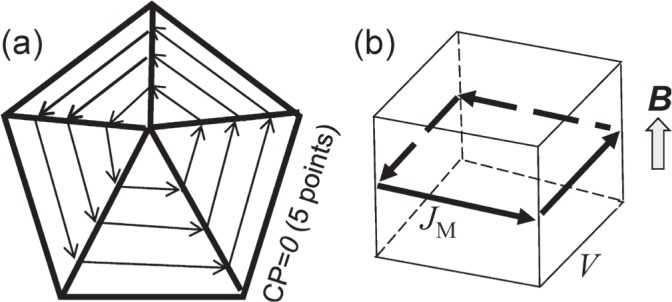
A computational model of a magnetic moment for a node current potential and magnetization currents on an iron piece surfaces creating a magnetic moment. (**a**) Current potential and mesh currents. (**b**) Iron piece and magnetization current *J*_M_.

This shimming calculation uses DUCAS^15^ which calculates the CP distribution according to a magnetic field distribution given by measurements. DUCAS forms the shim-trays by the triangle finite elements (FEs) and each node has a CP value *T*_i_ as Fig. 1 right. Calculated CP values are converted into MMs and iron piece volumes which generate ***B***^ER^ approximately. The CP value (described as *T*_j_ for the *j*-th node) denotes the magnetic moment density (A), because *T*_j_ is a value of the current flowing around the node^21^ as indicated in Fig. 4a. Since DUCAS uses triangle FEs, any surfaces for shim-trays can be treated. This study focuses on planar shim-trays as Fig. 1.

A node with *T*_j_ has a MM of,

(3) $$M_{j}=\sum_{k}S_{\text{kj}}n_{\text{k}}T_{\text{j}}/3,$$

where the summation is done over the *k*-th FE which includes the *j*-th node, and *S*_kj_ and ***n***_k_ are the area and normal vector of the *k*-th FE. The factor 1/3 is due to the triangle FE shape. As Fig. 1 shows, ***n***_k_ and ***M***_j_ are in the *Z* direction parallel to the magnetic field. Figure 4b shows magnetization current *J*_M_ on iron piece surfaces. The current generates a MM in the magnetic field direction (*Z*). Then, the two MMs in Fig. 4 are identical to each other and the CP distribution can be converted into the MM distribution and then the iron piece placement.

Using Eq. (2) and Eq. (3), we can describe the magnetic field distribution ***B***^MM^ due to CPs as,

(4) $$B^{\text{MM}}=AT,$$

where *A*_ij_ of the response matrix **A** is,

(5) $$A_{\text{ij}}=\left\{\mu_{0}/\left(4\pi r_{\text{ij}}^{3}\right)\right\}\left(3r_{\text{Zij}}^{2}/r_{\text{ij}}^{2}-1\right)\sum_{\text{k}}S_{\text{kj}}/3,$$

and ***T*** is a vector for which components are node CP values *T*_j_. The summation in Eq. (5) is done over the *k*-th FE which includes the *j*-th node.

The magnetic field after the shimming work becomes ***B***^MM^ + ***B***^MS^ which all node CP values should satisfy,

(6) $$-\varepsilon B_{\text{i}}^{0}<\left(B_{\text{i}}^{\text{ER}}+B_{\text{i}}^{\text{MM}}\right)<+\varepsilon B_{\text{i}}^{0},$$

where *ɛ* is an allowable residual rate and is half of the homogeneity (on the order of 10.0 × 10^−6^ or 10 ppm) on MFEPs or the VOI surface of the 40 cm-DSV in this study. In this shimming calculation, ***B***^MM^ is generated by CPs on the shim-trays ***T***^SH^ as,

(7) $$B^{\text{MM}}=AT^{\text{SH}}.$$

The shimming calculation must obtains ***T***^SH^, with which magnetic field ***B***^MM^ Eq. (6) is satisfied, then ***B***^MM^ is roughly equal to ***B***^ER^. DUCAS solves the equation to obtain such ***B***^MM^,

(8) $$T^{\text{SH}’}={\left(\mathrm{AR}\right)}^{*}B^{\text{ER}},$$

where ***R*** is a matrix to choose independent nodes,^15,20,21^ which CPs form ***T ***^SH’^(=***RT***^SH^), and the superscript * means a pseudo-inverse matrix. For the shimming calculation, all MFEPs have the same weights.

TSVD is applied in order to get a pseudo-inverse of **AR**. Then, SVD describes the matrix as,

(9) $$AR=\sum_{\text{i}=1}^{M_{D}}u_{\text{i}}\lambda_{\text{i}}\nu_{\text{i}}^{\text{t}},$$

where the summation is done over eigenmodes from *i*=1 to a truncation number *M*_D_ of TSVD regularization. The pseudo-inverse matrix can be calculated as,

(10) $${\left(\mathrm{AR}\right)}^{*}=\sum_{i=1}^{M_{D}}\nu_{i}u_{\text{i}}^{\text{t}}/\lambda_{\text{i}},$$

where ***v***_i_ is the *i*-th normalized eigenvector for ***T***, ***u***_i_ is the *i*-th normalized eigenvector for the magnetic field distribution, λ_i_ is a singular value, and the summation is over eigenmode number *i*. Eq. (8) and Eq. (10) yield,

(11) $$T^{\text{SH}}=R\sum_{i=1}^{M_{D}}N_{E}^{\frac{1}{2}}\nu_{i}D_{i}/\lambda_{i},$$

where *N*_E_ is the number of MFEPs and the *D*_i_ is an eigenmode strength for the error field, calculated by,

(12) $$D_{i}=u_{i}^{t}B^{\text{ER}}/N_{\text{E}}^{1/2}.$$

The summation in Eq. (11) is done over eigenmodes with a truncation as follows.

Since ***u***_i_ have a unity norm, *D*_i_ is a square averaged magnitude of the *i*-th eigenmode component of ***B***^ER^. The summation in Eq. (11) can be summed up to the rank **AR**, but generally, the high order (small *λ*_i_) eigenmodes produce a very weak magnetic field and a summation of a limited number of low order eigenmodes can reconstruct a sufficiently accurate magnetic field for ***B***^0^. The reconstructed magnetic field distribution ***B***^RC^ is obtained as,

(13) $$B^{\text{RC}}\left(M_{D}\right)=\sum_{i=1}^{M_{D}}N_{\text{E}}^{\frac{1}{2}}u_{i}D_{i}$$

The accuracy of ***B***^RC^ is a function of *M*_D_ and large *M*_D_ makes ***B***^RC^(*M*_D_) approach ***B***^0^, or it is important to choose *M*_D_ to get a good shimming result. The prediction of a residual magnetic field ***B***^RE^,

(14) $$B^{\text{RE}}=B^{0}-B^{\text{RC}}\left(M_{\text{D}}\right),$$

is mainly due to the eigenmodes which are not summed in Eq. (13). The shimming calculation predicts the peak-to-peak homogeneity *h* as,

(15) $$\begin{array}{l} h\left(M_{\text{D}}\right)=B_{\text{PP}}^{\text{RE}}\left(M_{\text{D}}\right)/B^{0}=\\ \;\;\;\;\;\;\;\;\left\{B_{\text{mx}}^{\text{RE}}\left(M_{\text{D}}\right)-B_{\text{mn}}^{\text{RE}}\left(M_{\text{D}}\right)\right\}/B^{0}[\text{ppm}], \end{array}$$

where $B_{\text{mx}}^{\text{RE}}$ and $B_{\text{mn}}^{\text{RE}}$ are maximum and minimum residual magnetic field on MFEPs. ***B***^RE^ of Eq. (14) has two components. The truncation error of Eq. (13), or the magnetic field from the higher ordered eigenmodes than *M*_D_, is a main component on $B_{\text{PP}}^{\text{RE}}\left(M_{\text{D}}\right)$ and is larger than several μT. A measurement error is an additional and weak component less than 0.1 μT. Then, *h*(*M*_D_) is almost determined by the truncation error.

Since MFEPs are on the 40 cm-DSV, the homogeneity on 40 cm-DSV is denoted by *h*_40_, which is used not only for the measured ***B***^ER^ but also for the predicted residual ***B***^RE^. The calculated *h*_40_(*M*_D_) is expected to approach zero by increasing *M*_D_ with decreased truncation error in Eq. (13), and *h*_40_(*M*_D_) predicts the homogeneity after the shimming work. The shimming calculation determines *M*_D_ which satisfies

(16) $$h_{40}\left(M_{\text{D}}\right)<2\varepsilon .$$

On the other hand, *h*_40_(0) is without a shimming work and is a homogeneity for the measured magnetic field ***B***^MS^. In this paper, *h*_40_(0) is simplified as *h*_40_ and it is usually described by the unit of ppm (1 × 10^−6^).

### Input and output parameters

The shimming calculation needs two input parameters of target magnetic field *B*^0^ and the truncation number *M*_D_ which is the upper most eigenmode included in the shimming calculation {Eq. (11) and (13)}, other than measured magnetic field data. The output parameters are CP distribution ***T***^SH^ of Eq. (11), predicted residual magnetic field ***B***^RE^ of Eq. (14) and predicted homogeneity {for example, *h*_40_(*M*_D_) of Eq. (16)}. Eq. (14) shows that these outputs depend on two input parameters of *B*^0^ and *M*_D_.

The expected homogeneity directly depends on *M*_D_. Increase of *M*_D_ improves homogeneity, but increase the total iron volume. Too large *M*_D_ may make the iron volume too large and iron piece placements impossible. Since the homogeneity is usually monotonically improved as *M*_D_ increases, *M*_D_ should be determined at a value with which homogeneity meets the specification.

On the other hand, *B*^0^ has an optimum value near the average magnetic field strength of the measured magnetic field <*B*^MS^>. *B*^0^ magnitude is to be set as <*B*^MS^> as a first candidate and it is tuned to get good homogeneity with reasonable iron volumes. The tuning of *B*^0^ is done using this shimming calculation, so that predicted homogeneity meets the specification with adequate iron volume placements.

### Iron piece placements

#### Iron volume distribution

In order to obtain the iron piece placements as shims, ***T***^SH^ of Eq. (11) is converted into the iron volume distribution, as follows. An iron piece in a magnetic field of the MRI magnet is almost magnetically saturated at magnetization 2.15T as reported in Refs. (9, 11). The magnetization corresponds to a surface current *J*_M_ (Fig. 4b) of 1.711 × 10^6^ A/m and magnetic moment of 1.711 × 10^6^ Am^2^ per unit volume of 1.0 m^3^ on the shim-tray. Each element of ***T***^SH^ is converted into iron volume. For example *V*_j_ of *j*-th node is,

(17) $$\begin{array}{l} V_{\text{j}}=M_{\text{Zj}}/1.711\times 10^{6}=\left\{{\sum_{\text{k}}S}_{\text{kj}}T_{\text{J}}^{\text{SH}}/3\right\}\\ \;\;\;\;\;\;\;\;\;\;\;\;\;\;\;\;\;\;\;\;\;\;\;\;\;\;\;\;\;\;\;\;\;\;\;\;\;\;\;\;\;\;\;\;\;\;\;\;\;\;\;\;\;\;\;\;\;\;\;\;/1.711\times 10^{6}\left[{\text{m}}^{3}\right], \end{array}$$

where subscript *j* denotes the *j*-th node on the shim-trays of Fig. 1, *V*_j_ is a function of *M*_D_ through Eq. (11) and the summation in Eq. (17) is done over the *k*-th FE which includes the *j*-th node. Usually, the number of the shim-tray nodes is large as several thousands in Fig. 1 and *V*_j_ values are summed in areas of a few tens of cm^2^ on the trays to get definite iron mass. Since ***T***^SH^ is obtained through Eq. (11) of summation on eigenmodes, *V*_j_ of Eq. (17) has contributions from selected eigenmodes which number is less than *M*_D_ and will be placed on the two shim-trays of top and bottom. We have to design the shim-trays so that the iron pieces calculated by Eq. (17) are possible to be placed. The design baselines are as follows.

### Shim-tray size and expression of eigenmodes

The shim-trays should be designed to have a capability to shim the magnetic field to make the homogeneity less than the specification. We should have two considerations. One is eigenmodes and another is volumes of iron pieces.

About the eigenmodes, following considerations were applied. The shim-trays (Fig. 1) should represent the distributions of eigenmodes ***v***_i_, which are necessary for the homogeneous magnetic field and are up to *M*_D_-th. Such eigenmodes were discussed in Refs. (3, 4), which developed a magnet design method with two dimensional (2D) SVD eigenmodes with a symmetry in axial (*Z*) direction. They show that only six 2D symmetry eigenmodes are fully and the 7-th 2D eigenmode is fractionally used for usual MRI magnet with 6 main CBs. Then, the shim-trays are considered to have the capability to reconstruct up to the 7-th 2D symmetry eigenmode. In the following part of this paper, such symmetric eigenmodes are called as the basic eigenmodes.

The capability to represent the eigenmodes depends on *R*_S_/*Z*_S_ and FE sizes of the computational models in the shimming calculation. Shim-trays with large *R*_S_/*Z*_S_ can represent a larger number of eigenmodes and high ordered SVD eigenmodes need small size fine FEs in the computational model.

About the iron volumes, we have to design the shim-trays so that they have the capabilities to hold enough iron volumes to compensate the error fields. During the magnet design, we estimate possible error fields and evaluate iron piece placements by the shimming calculations. Then, we have to design the shim-trays which have enough volume capabilities to compensate the error fields.

The designed shim-trays and capabilities are discussed and confirmed with real magnetic field data of the test magnet a in the section **Results** in later.

### Negative volumes in shimming calculation result and repetitive work

In the geometry of open MRI magnet like Fig. 1, the magnetic field due to MMs on the shim-trays are mainly positive as indicated in Fig. 3 and this magnetic fields strengthens the magnetic fields in FOV. Then, *B*^0^ is set to be a slightly higher magnetic field than the measured average one to reduce the negative iron volume.

**Fig 5.**
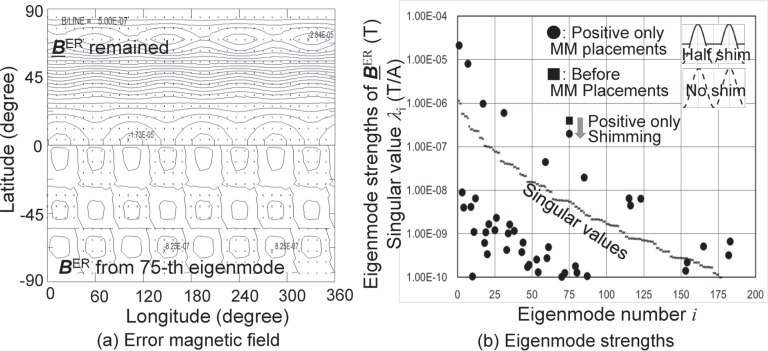
Computational shimming test for 75-th eigenmode of 0.4 μT strength or 1.65 μT peak-to-peak. (**a**) Assumed error magnetic field distribution in the bottom half and remained error magnetic field distribution after positive only MM shimming in the top half. Contour lines are at every 0.5 μT. (**b**) Eigenmode strengths of the error magnetic field. A black square denotes the strengths before the shimming ***B***^ER^ and black circles denote those remained error magnetic field ***Ḇ***^ER^ after the positive only shimming. The placements are illustrated in small frames at the top right position. MM, magnetic moment.

Sometimes, the calculation yields negative *V*_i_. This situation is seen especially for cases with lower *B*^0^ than averaged one and/or too large *M*_D_ number, which includes high ordered eigenmodes. They are likely to have large amplitudes CP distributions because of the small singular values and they easily lead to yield negative *V*_i_ values. Permanent magnet pieces may be applicable for the negative *V*_i_, however, it is possible to get the shimmed magnetic field even with only passive iron pieces. The possible technique is to place iron pieces only at the positive area and to repeat the shimming work as explained roughly in Fig. 5, in which a computational test shimming is described and ***B***^ER^ is assumed to have only the 75-th eigenmode distribution, i.e. ***B***^ER^ = *N*_E_^1/2^***u***_75_*D*_75_ with *D*_75_ = 0.4 μT.

In Fig. 5a, bottom half plots the contour lines for the ***B***^ER^ distribution and top half plots those for the remained error field ***Ḇ***^ER^,

(18) $${\underline{B}}^{\text{ER}}=A\left\{\left(\nu_{\text{i}}-{\underline{\nu }}_{\text{i}}\right)\left(u_{\text{i}}^{\text{t}}B^{\text{ER}}\right)/\lambda_{\text{i}}\right\},$$

after the positive only iron piece placements, where ***v̱***_i_ is the distribution obtained from ***v***_i_ with making the negative elements forced zero. Figure 5b shows eigenmode strengths of ***Ḇ***^ER^. The error field amplitude (then homogeneity) is increased by this iron piece placement. However, this situation shows a shimming progress. The eigenmode strengths for ***B***^ER^ (square) before and ***Ḇ***^ER^ (circle) after the positive only shimming are plotted in Fig. 5b. Due to the shimming, 75-th eigenmode strength is reduced by half, while some other eigenmodes appear. Those in higher eigenmode numbers than 75-th (original) are less than 1/10 of the original 75-th strength, meaning that they are negligible. Those in low eigenmode number are larger than the original 75-th strength. However, they can be easily shimmed with small volumes of iron pieces, because of large singular values for them as plotted in Fig. 5b.

As a summary of the discussion so far, we can expect that the shimming without the negative iron piece placements, can shim the error magnetic field as reduced eigenmode strengths. However, the strengths at low eigenmode numbers may be increased. In order to make these low ordered eigenmodes shimmed, two points should be considered. One is that the repetitive shimming work is necessary, and the other is that *B*^0^ should be tuned considering that the magnetic fields on MFES and in VOI are generally strengthened due to the placed iron pieces. In the test shimming of the next section, this technique of positive only shimming the repetition and *B*^0^ tuning were adopted.

The shimming procedure needs some repetitions as shown in Fig. 2 until the homogeneity meets the specification. Cause of the repetitive work is considered to be due to errors of the iron piece volumes placed and the errors from the ignored negative volumes is the largest among iron volume error sources. There are two possible error iron volume sources other than the ignored negative volumes. They are iron piece tolerances and magnetization strengths of iron pieces. The first one depends on shimming calculation parameters and the errors are estimated to reach 30% of calculated volumes at maximum. The others are estimated to be less than 10% of calculated volumes. These may increase the number of the repetitions, while the homogeneity is expected to improve repetitively to meet the specification. This shimming calculation with the repetitive shimming work were confirmed experimentally on shimming works as next sections.

## Results

Test shimming works were done to confirm the applicability of the developed shimming calculation method to MRI magnets. The target homogeneity in the following test shimming was decided as 10 ppm in VOI of 40 cm-DSV on a 0.5T open type MRI magnet (*h*_40_ = 10 ppm). The homogeneity 10 ppm corresponds to $B_{\text{PP}}^{\text{RE}}=5\;\mu \text{T}$ and has the following meanings.

1. $B_{\text{PP}}^{\text{RE}}=5\;\mu \text{T}$ corresponds 3.3 ppm for 1.5T magnet, meaning a high field magnet can be shimmed down to a few ppm by this method.
2. With *h*_40_ = 10 ppm, an MR image has little distortion (0.5 mm distortion in 40 cm-DSV) due to *B*^RE^ of −2.5 to 2.5 μT, even with weak gradient field of 5 mT/m, which is 1/5 of the maximum gradient field strength in this system.

### Test shimming geometry

The shimming calculation method described in the former section was applied to test MRI magnet shimming works. The magnet was an open type (Fig. 1) with 0.5T magnetic field strength. The iron pieces were placed 3 cm away from pole surfaces, and they were considered to be at saturated magnetization 2.15T.^11^ The shim-trays, in which iron pieces were placed according to the shimming calculation results, were *R*_S_ = 0.54 m radius thin planar shapes placed at *Z* = +0.339 m and *Z* = −0.339 m. The FE sizes of computational models were determined to be less than 2.5 cm, and actual sizes are 0.8 cm (center) to 2.0 cm (edge) and the total number of nodes on the computational model of shim-trays is 5282. Maximum iron volume density on the trays was designed as 0.25 cm^3^/cm^2^, which corresponded to the maximum CP value of 5.13 kA. There were several kinds of iron piece volumes of 0.04 to 1.0 cm^3^ and the pieces were placed within 0.329 m < |*Z*| < 0.349 m or 2 cm thickness.

There were 768 MFEPs on the MFES with 24 points along the latitude angle and 32 planes in the longitude angle. MFEPs were at the same position as the MFMPs and on the surface of VOI (40 cm-DSV). Homogeneity *h*_40_ was calculated from peak-to-peak magnetic field strength amplitude at MFEPs.

### SVD eigenmodes

**Fig 6.**
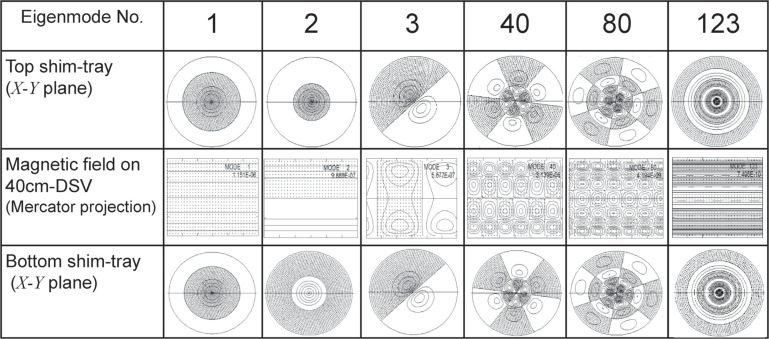
Examples of eigenmodes in the shimming calculation obtained by singular value decomposition (SVD). Six eigenmodes are plotted as examples from identified 482 eigenmodes. Among the examples, the first and 123-th eigenmodes are basic eigenmodes which magnetic distributions have two dimensional (2D) (azimuthal and *Z*) symmetries.

The response matrix **A** had a size of 768 × 5282 and the number of identified eigenmodes were 482. Among them, sample six eigenmodes are shown in Fig. 6. Contour lines of CP distributions on the top and bottom shim-trays and of magnetic field distributions on the MFES are plotted for six sample eigenmodes from the identified 482 eigenmodes. They are three low ordered (large *λ*_i_ value) eigenmodes (1-st, 2-nd and 3-rd) and three middle to high ordered eigenmodes (40-th, 80-th and 123-th). Dotted areas have negative values.

The first and the 123-th eigenmodes are 2D up-down symmetry and are the basic eigenmodes. The No. 6 and No. 7 basic eigenmodes^1,2^ are at the 85-th and 123-rd eigenmodes, respectively, in this shimming geometry. Increasing the order (decreasing *λ*_i_ values), the eigenmode distributions become fine and they can reconstruct the detailed magnetic field distribution with large CP (then, heavy iron piece volume) values. The high ordered (higher than the 85-th) eigenmodes are chosen when a magnetic field shimming with fine magnetic field distribution is necessary. *M*_D_ in Eqs. (11), (13) and (14) is determined to get a good homogeneity and the basic eigenmodes are available as markers.

### Test shimming work results

**Fig 7.**
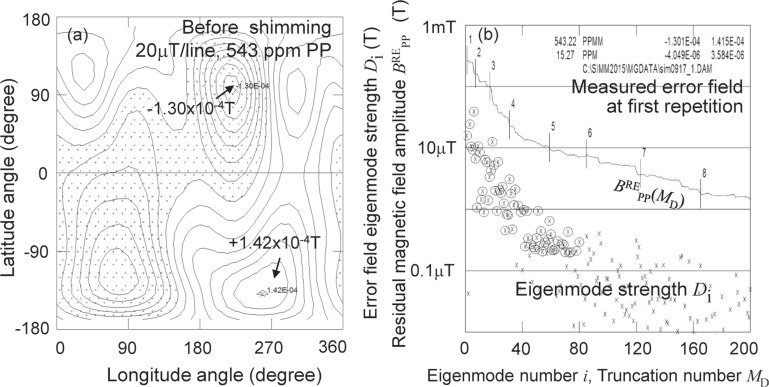
Error magnetic field before shimming on 40 cm-DSV surface. (**a**) Measured error magnetic field distribution with contour lines at every 2.0 × 10^−5^T. For the dotted area, *B*^ER^ < 0 (*B*^MS^ > *B*^0^). (**b**) Eigenmode strengths (crosses) and predicted peak-to-peak amplitude $B_{\text{PP}}^{\text{RE}}\left(M_{\text{D}}\right)$ (line). The eigenmodes plotted with circles are selected as those to be shimmed. The numbered short lines denote the basic eigenmodes. Numbers at the top right are the measured and predicted *h*_40_ values with $B_{{\text{mn}}^{\prime }}^{\text{ER}}$, $B_{\text{mx}}^{\text{RE}}$ and $B_{{\text{mn}}^{\prime }}^{\text{ER}}$, $B_{\text{mx}}^{\text{RE}}$. The shimming calculation predicted that *h*_40_ = 15.27 ppm could be attainable by the iron piece placements obtained by this shimming calculation with *M*_D_ = 80.

Figure 7a shows ***B***^ER^ calculated by Eq. (1) with *B*^0^ = 0.499998 T. This *B*^0^ was determined at average <*B*^MS^> and *h*_40_ was 543 ppm. Contour lines are plotted on the Mercator projection at MFES (40 cm-DSV). In dotted area, the measured magnetic fields exceed *B*^0^ and *B*^ER^ is negative.

Figure 7b plots the eigenmode strengths *D*_i_ of Eq. (12) and $B_{\text{PP}}^{\text{RE}}\left(M_{\text{D}}\right)$ calculated from Eqs. (14) and (15) for Fig. 7a ***B***^ER^, as a function of *M*_D_. The circled eigenmodes were chosen to be shimmed with the limitation of *D*_i_ > 0.2 μT which limitation was not necessary for usual shimming, but this was a test and we included it to confirm that the eigenmodes were well controlled. When chosen eigenmodes are well shimmed as Eq. (13) and (14) for *i* < *M*_D_, the predicted *h*_40_(80) from Eq. (15) is 15.3 ppm ($B_{\text{PP}}^{\text{RE}}(80)=7.6\;\mu \text{T}$). Short vertical lines with numbers indicate the basic eigenmodes. The chosen *M*_D_ for the shimming are consistent with up to the 6-th basic eigenmodes. The expected *h*_40_ = 15.3 ppm was a little larger than the target homogeneity *h*_40_ = 10 ppm, but this first test shimming work was done to confirm the validity of this shimming calculation. A shimming for the target homogeneity will be discussed after this.

**Fig 8.**
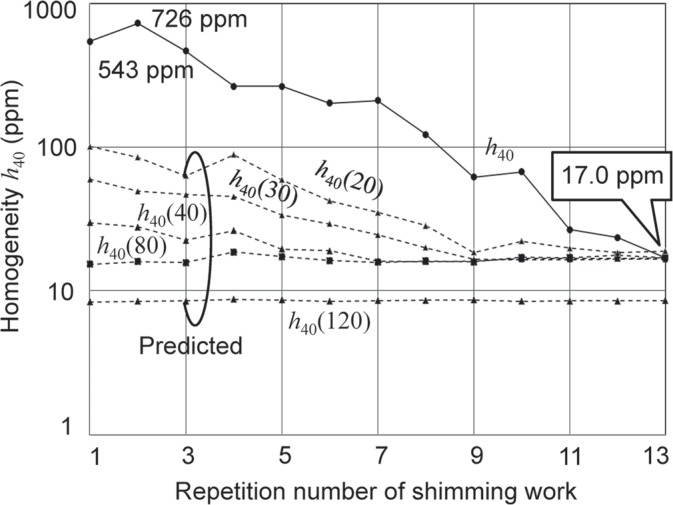
Homogeneities during the test repetitive shimming work. The top line is for the measured and obtained homogeneity *h*_40_. The rests are predicted homogeneities *h*_40_(*M*_D_) from the residual magnetic fields calculated by this shimming calculation with *M*_D_ = 20 to 120.

Figure 8 shows the obtained (solid line) *h*_40_ and predicted *h*_40_(*M*_D_) (dashed lines) plotted by functions of repetition. They finally obtained *h*_40_ was 17.0 ppm which was quite near the predicted *h*_40_(80) of 15.3 ppm in Fig. 7. The dashed lines are *h*_40_(*M*_D_) calculated by Eq. (15) with Eq. (13) and Eq. (14). If the eigenmodes up to *M*_D_ are ideally shimmed (*D*_i_ = 0 for *i* < *M*_D_), the predicted *h*_40_(*M*_D_) can be obtained as the measured one i. e. that is *h*_40_(0) after the shimming. However, the iron piece placements are estimated to have roughly 30% volume errors in this test shimming work. The errors are the cause that the repetition is necessary for the shimming work. *D*_i_ values at *i* = 50 to 80 have to be shimmed to reduce *D*_i_ by 1/10 roughly. Since the shimming is considered to be done with the errors randomly, at least four repetitions are necessary to shim the eigenmodes. Around the 40-th eigenmode, *D*_40_ should be shimmed as 1/30 and at least six repetitive works are necessary. For the 1 to 10-th eigenmodes, at least 12 repetitions are necessary to reduce *D*_i_ by 1/1000 for the test shimming works. Then it can be estimated that the number of repetition more than 12 times may be necessary in the test shimming works.

The test shimming work was done with *M*_D_ = 80 in Fig. 7. This is why *h*_40_(80) had a little change meaning that the *D*_i_ values for *i* > 80 were not shimmed during the repetitive shimming work. For the same reason, *h*_40_(120) had little change during the repetitive work. However, the others {*h*_40_(*M*_D_= 0 to 40)} were reduced repetitively. At the second repetition (after the first iron placements), *h*_40_ was deteriorated, while the *h*_40_(*M*_D_ = 20 to 40) values were reduced, meaning that the shimming was effective for the eigenmodes of *i* = 20 to 80 in the first repetition. We have to check the eigenmode strength *D*_i_ as well as homogeneity itself (*h*_40_) during the repetitive shimming work.

**Fig 9.**
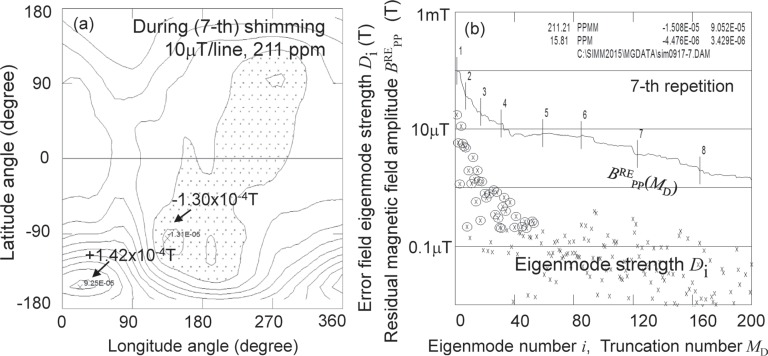
Error magnetic field and its eigenmode strengths at the 7-th shimming calculation during repetitive shimming work. (**a**) Error magnetic field distribution on magnetic field evaluation surface (MFES) (40 cm-DSV) with contour lines at every 1.0 × 10^−5^T. For the dotted area, *B*^ER^ < 0 (*B*^MS^ > *B*^0^). (**b**) Eigenmode strengths *D*_i_ (crosses) and predicted peak-to-peak (PP) amplitude $B_{\text{PP}}^{\text{RE}}$ of the residual magnetic field (line) as functions of *M*_D_. The eigenmodes plotted with circles are selected as the ones to be shimmed. The numbered short lines denote that they are basic eigenmodes. Numbers at the top right are the measured and predicted *h*_40_ values with $B_{\text{mn}}^{\text{ER}}$, $B_{\text{mx}}^{\text{ER}}$ and $B_{\text{mn}}^{\text{RE}}$, $B_{\text{mx}}^{\text{RE}}$. At *M*_D_ = 80, *h*_40_(80) = 15.8 ppm is predicted.

Figure 9 plots (a) error magnetic field ***B***^ER^ and (b) *D*_i_ values and a line for the $B_{\text{PP}}^{\text{RE}}\left(M_{\text{D}}\right)$ at 7-th repetitive work. The homogeneity was 211 ppm with a reduced number of peaks as shown in Fig. 9a. In Fig. 9b, the 50-th to 80-th eigenmodes had small *D*_i_ values, less than 0.2 μT as intended. They were shimmed from the values in Fig. 7b. The $B_{\text{PP}}^{\text{RE}}\left(M_{\text{D}}\right)$ line plot is flat between *M*_D_ of 50 to 80 because of small *D*_i_ values for *i* = 50 to 80. These plots show that, during the repetitions (first to 7-th), 50-th to 80-th eigenmodes were well shimmed. It can be recognized that the shimming is done from high ordered to low ordered eigenmodes during the repetitive shimming work and this characteristic is consistent with Fig. 5.

**Fig 10.**
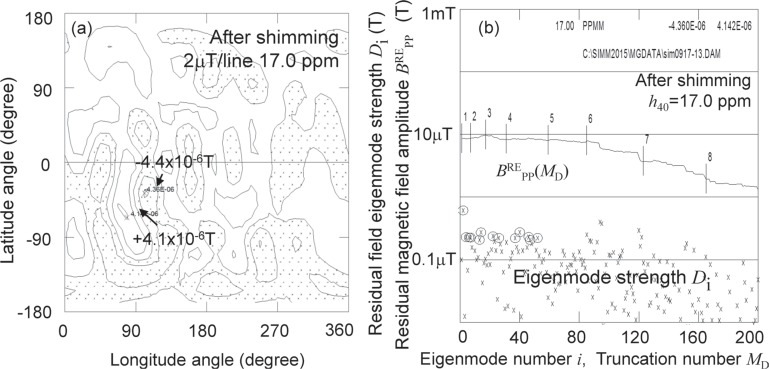
Residual magnetic field after the 1-st test shimming work finish. The homogeneity is *h*_40_ = 17.0 ppm. (**a**) Residual magnetic field distribution with contour lines at every 2.0 μT. For the dotted area, *B*^ER^ < 0 (*B*^MS^ > *B*^0^). (**b**) Eigenmode strengths and predicted peak-to-peak (PP) amplitude $B_{\text{PP}}^{\text{RE}}$ of the residual magnetic field (line) as functions of *M*_D_. The numbered short lines denote that they are basic eigenmodes. At *M*_D_ = 80. *h*_40_ = 17.0 ppm is predicted, meaning that no further shimming is applicable.

Figure 10a shows a ***B***^RE^ after shimming finished and $B_{\text{PP}}^{\text{RE}}$ is 8.5 μT which has been greatly reduced from 272 μT in ***B***^ER^ before the shimming started. Figure 10b plots *D*_i_ and $B_{\text{PP}}^{\text{RE}}$. The *D*_i_ values have been shimmed and reduced compared to Fig. 7b and the $B_{\text{PP}}^{\text{RE}}\left(M_{\text{D}}\right)$ line is flat at *M*_D_ < 80. These show that the repetitive shimming work has finished and this first test shimming work has confirmed that the passive shimming with this novel shimming calculation works well.

Since our target homogeneity was *h*_40_ = 10 ppm, we could understand from Fig. 10b, shimming including higher ordered eigenmodes than 80-th and reduction of *D*_i_ (*i* < 80) less than 0.2 μT were necessary. Another test shimming was done using the same magnet. In order to obtain *h*_40_ less than 10 ppm, following two policies were introduced.

1. The shimming for the eigenmodes up to 160-th were added to make *D*_i_ of high ordered (80 to 160-th) eigenmodes small before the first repetition.
2. During the repetition, the eigenmode strengths were shimmed to be *D*_i_ < 0.05 μT instead of 0.2 μT of 1-st test shimming.

**Fig 11.**
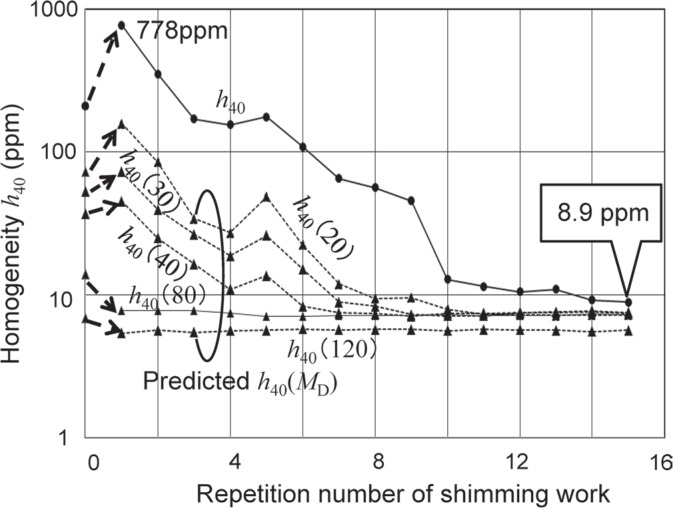
Homogeneities during the repetitive shimming work of the 2-nd test shimming. The top line data are for obtained homogeneity *h*_40_. The rest of the data are predicted *h*_40_(*M*_D_) by the shimming calculations with *M*_D_ = 20 to 120. Before this test shimming, eigenmodes of number 80-th to 120-th are roughly shimmed as shown by the 0-th to 1-st repetitive shimming work to obtain a better homogeneity than the first test shimming.

Figure 11 plots the results. As shown by arrows with dashed lines, *h*_40_(*M*_D_ = 80) and *h*_40_(*M*_D_ = 120) were reduced before the first repetition. Roughly the same characteristics were observed as the former test shimming after the first repetition.

**Fig 12.**
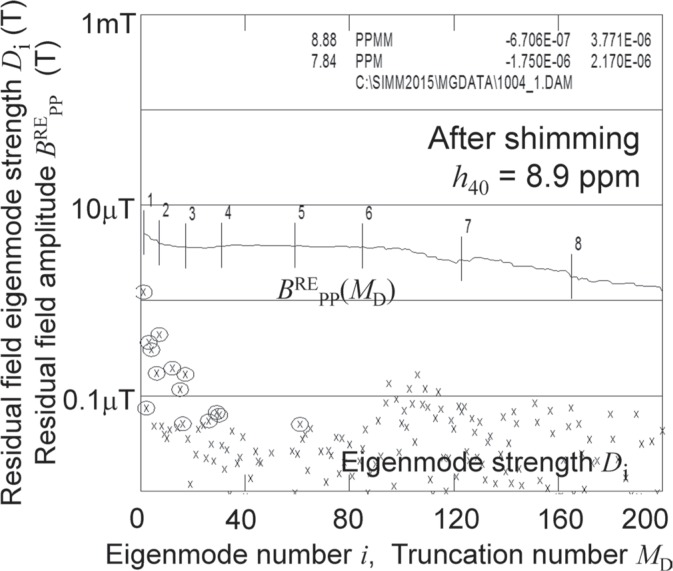
Eigenmode strengths for the residual magnetic field at the 15-th shimming calculation after the repetitive shimming work. Measured homogeneity is *h*_40_ = 8.9 ppm and predicted homogeneity *h*_40_(*M*_D_) are shown as the line. The numbered vertical short lines denote the basic eigenmodes. The calculation predicts that slightly improved homogeneity *h*_40_ = 7.8 ppm could be obtained with *M*_D_ = 80 with further repetitive shimming work.

Figure 12 plots *D*_i_ of Eq. (12) for 15-th repetition. Calculated $B_{\text{PP}}^{\text{RE}}\left(M_{\text{D}}\right)$ is also plotted as a line and *h*_40_ = 8.9 ppm at *M*_D_ = 0. At the same time the predicted *h*_40_(80) was 7.8 ppm, meaning that the repetitive shimming work could be continued to get better *h*_40_. However, this test shimming was terminated at 15-th repetitive work, because a better *h*_40_ than the first test shimming work and less than 10 ppm has been obtained with the reduced *D*_i_ of the 80-th to 160-th eigenmodes and less than 0.05 μT *D*_i_ eigenmode strengths at 20-th to 80-th as intended. The difference, between the obtained *h*_40_ = 8.9 ppm and predicted *h*_40_(80) = 7.8 ppm, is considered to be due to the low ordered eigenmodes (*i* < 20) plotted with circles.

The test shimming results obtained were very promising. Since then, this shimming calculation has been applied to installations and maintenances of open type MRI magnets up to 1.2T. With this method, the shimming works are usually done in one day.

## Discussion

These two sets of test shimming results have shown that the shimming calculation method in this study works well. Here, we discuss two subjects: the calculated negative iron volumes and the advantages of this method. These discussions suggest that this method has possibilities of extended applicability.

### Calculated negative iron volume

**Fig 13.**
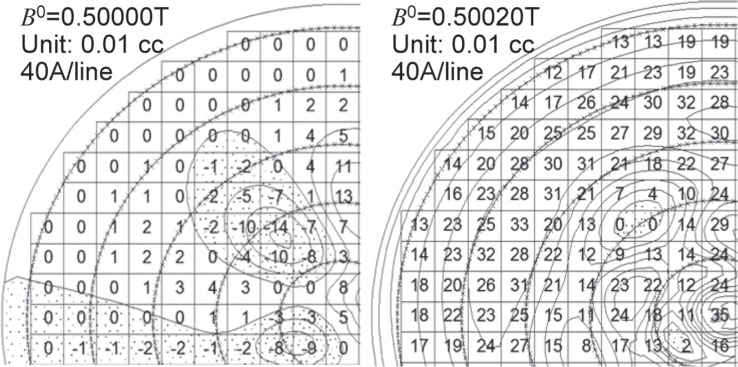
Iron piece volume and current potential distributions on a bottom shim-tray with unit of 0.01 cc per 25 cm^2^ area and contour lines at every 40A, calculated by the shimming calculations at 7-th repetitive shimming work for two *B*^0^. Circles are plotted to make the radius position clear at radiuses of 10, 20, 30, 40 and 50 cm.

The shimming calculation sometimes yields negative volume iron piece placements partially and a technique to deal with them was discussed with Fig. 5, which suggested repetitive shimming work was necessary. Another possible technique is considered from Fig. 3, which shows that the iron pieces on the shim-tray generate a positive magnetic field. Then, to delete the negative calculated iron volume, an increased *B*^0^ may be adequate. Fig. 13 is an example which shows iron volumes per 25 cm^2^ at the bottom shim-tray of a 1/4 part. The unit of the numbers is 0.01 cm^3^. The left side is calculated at *B*^0^ = 0.50000T and the right is at *B*^0^ = 0.50020T. At *B*^0^ = 0.50000T, some negative iron volumes were calculated. At *B*^0^ = 0.50020T, no negative numbers were calculated. This comparison clearly shows that increasing *B*^0^ is effective to eliminate calculated negative iron volumes. However, the right side also shows that increased *B*^0^ needs a larger iron volumes than low *B*^0^. We have to choose *B*^0^ which deletes the negative iron volumes during the repetition without too much increase of the iron volumes and this shimming calculation is available to choose the adequate *B*^0^.

### Advantage of TSVD in shimming calculation

The first advantage of the TSVD is that the computational time does not disturb the shimming work. Since the geometry is fixed for one model series of MRIs and the SVD calculation is only done once in advance, the computational time for the shimming calculation at a site is quite short. One calculation, from reading the measured data, through summing SVD eigenmodes, to the iron piece placements, takes about 5 seconds with 5282 nodes, 768 MFEPs, and 3.3 GHz i5 CPU. This computational time enables the search for the optimum shimming condition for *B*^0^, *M*_D_ and attainable homogeneity.

The second advantage is that the eigenmodes included in Eq. (11) of the shimming calculation are selected from low order eigenmodes, which have large singular values, and magnetic fields are generated with small iron volumes. Then, this method has a capability to carry out the shimming works with small iron volumes. Some methods with LO^8,9,11,12^ use constraints of not only homogeneity but also shim amounts. The results depend on the weight factors. However, in our method we understand that the specified homogeneity can be obtained with a small shim amount.

The third advantage is that it is easy to understand the attainable homogeneity as a line of $B_{\text{PP}}^{\text{RE}}$ in Fig. 7b from the measured magnetic field. From the line, we can choose the shimming calculation parameter *M*_D_ easily. The eigenmodes included in the shimming calculation are considered to be related to magnet designs i.e. the number of main CBs.^3,4^ This understanding is based on the TSVD regularization and we can easily get attainable homogeneity along with the MRI magnets.

## Conclusion

A novel passive shimming calculation method has been developed and applied to an open MRI magnet. Shimming works are to correct the error magnetic fields to make magnetic fields homogeneous for the MR imaging. The shimming calculation calculates the MM distribution which generates the correction magnetic field and converts it into iron piece placements for the shimming works. The calculation is done from the measured error magnetic fields, which are the differences between the uniform target magnetic fields and the measured magnetic fields. The calculation method is based on TSVD regularization on a response matrix which describes the relation between the MM distributions (which is the CP distribution) on the shim-trays and the magnetic fields on MFEPs. The MM distributions are obtained through a superposition of the SVD eigenmodes with truncation.

Shimming tests were done on a 0.5T MRI magnet. It have been concluded the novel shimming calculation method is well applicable to MRI magnets shimming works with adequate target magnetic field *B*^0^ and truncation eigenmode number *M*_D_.
